# TAL effectors and the executor *R* genes

**DOI:** 10.3389/fpls.2015.00641

**Published:** 2015-08-20

**Authors:** Junli Zhang, Zhongchao Yin, Frank White

**Affiliations:** ^1^Department of Plant Pathology, Kansas State University, Manhattan, KS, USA; ^2^Temasek Life Sciences Laboratory, National University of Singapore, Singapore, Singapore; ^3^Department of Plant Pathology, University of Florida, Gainesville, FL, USA

**Keywords:** TAL effectors, *R* gene, *Xanthomonas*

## Abstract

Transcription activator-like (TAL) effectors are bacterial type III secretion proteins that function as transcription factors in plants during Xanthomonas/plant interactions, conditioning either host susceptibility and/or host resistance. Three types of TAL effector associated resistance (*R*) genes have been characterized—recessive, dominant non-transcriptional, and dominant TAL effector-dependent transcriptional based resistance. Here, we discuss the last type of *R* genes, whose functions are dependent on direct TAL effector binding to discrete effector binding elements in the promoters. Only five of the so-called executor *R* genes have been cloned, and commonalities are not clear. We have placed the protein products in two groups for conceptual purposes. Group 1 consists solely of the protein from pepper, BS3, which is predicted to have catalytic function on the basis of homology to a large conserved protein family. Group 2 consists of BS4C-R, XA27, XA10, and XA23, all of which are relatively short proteins from pepper or rice with multiple potential transmembrane domains. Group 2 members have low sequence similarity to proteins of unknown function in closely related species. Firm predictions await further experimentation on these interesting new members to the *R* gene repertoire, which have potential broad application in new strategies for disease resistance.

## Introduction

*Xanthomonas* infects monocotyledonous and dicotyledonous plant species, and the pathogenicity of many species depends in part on the effector proteins secreted by a type III secretion (T3S) system (Leyns et al., 1984; Tampakaki et al., 2004). The transcription activator-like (TAL) effector family is a distinct family of type III effectors, which includes members with cognate susceptibility (*S*) and/or resistance (*R*) genes. TAL effectors function as host gene specific transcription factors that can target both *S* and *R* genes, leading to enhanced expression and consequential phenotypic effects (Gu et al., 2005; Yang et al., 2006; Kay et al., 2007; Römer et al., 2007). Susceptibility (*S*) genes are genes with TAL effector-dependent expression and have measurable effects on disease symptoms (Yang et al., 2006; Boch et al., 2014). TAL effector genes are limited to members of the genus *Xanthomonas* and *Ralstonia* (Hopkins et al., 1992; De Feyter et al., 1993; Salanoubat et al., 2002; Heuer et al., 2007). The genes are ubiquitous in some species and have apparent critical functions in a number of diseases (Yang and White, 2004; Cernadas et al., 2014; Cohn et al., 2014; Hu et al., 2014; Schwartz et al., 2015).

Three types of TAL effector associated *R* genes have been reported-recessive, dominant non-transcriptional (classical) and dominant TAL effector-dependent transcriptional based resistance. TAL effector-dependent recessive resistance occurs in rice lines with DNA polymorphisms in *S* gene effector binding elements and will not be discussed in detail here (Hutin et al., 2015). Dominant non-transcriptional based resistance is represented solely by the NBS-LRR resistance gene from tomato, *Bs4*, which was identified as the cognate *R* gene to the TAL effector gene *avrBsP*/*avrBs4* (Bonas et al., 1993; Schornack et al., 2004). However, a transcriptionally functional TAL effector is not required for *Bs4* resistance elicitation as truncated versions of the cognate avirulence gene also trigger resistance. Here, we discuss the third type, namely, TAL effector-dependent *R* genes that are both direct targets of TAL effectors in the host and identified as *R* genes. The genes have been referred to as terminator or, here, executor *R* (*E*) genes (Bogdanove et al., 2010; Tian et al., 2014). *E* gene expression, like Avr/R gene interactions, is associated with hypersensitive response (HR) on the respective host plants and restricts pathogen growth at the site of infection. Five *E* genes and the cognate TAL effector genes have been cloned, including *Xa27*, *Bs3*, *Bs4C-R*, *Xa10*, and *Xa23* (Gu et al., 2005; Römer et al., 2007; Strauss et al., 2012; Tian et al., 2014; Wang et al., 2015). The TAL effector AvrXa7 may target an as yet uncharacterized *E* gene *Xa7* due to the requirements for the effector nuclear localization signals (NLSs) and the transcription acidic activator domain in *Xa7*-dependent resistance (Hopkins et al., 1992; Yang et al., 2000).

## *E* Gene Variation is in the Promoter

**FIGURE 1:**
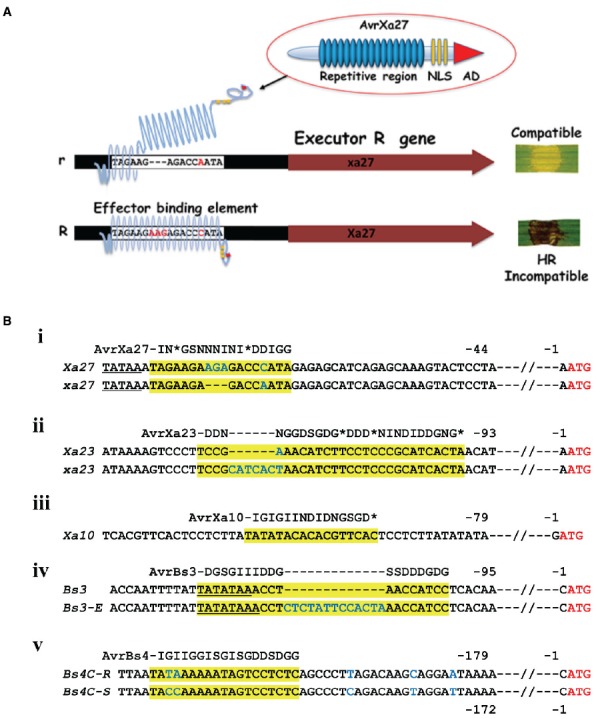
**TAL effector and ***E*** gene interactions. (A)** Schematic of the interaction between AvrXa27 and *Xa27*. Lower case r indicates the ineffective allele that lacks the AvrXa27 effector binding element. The r allele is missing three nucleotides and different in one nucleotide in the effector binding element of *Xa27* and does not permit binding of AvrXa7 leading to a compatible interaction (Römer et al., 2010). R indicates the dominant and functional allele of *Xa27*. *Xa27* expression leads to a resistance response and HR on leaves, indicated by the dark discoloring of the inoculation site (here, on a rice leaf). NLS, nuclear localization signal. AD, transcription activation domain of TAL effector. **(B)** Promoters of *E* genes and polymorphisms in dominant and recessive alleles. (i) Sequence alignment of a part of the promoters of *Xa27* from the rice cultivar IRBB27 (gi 66735941 gb AY986491.1) and *xa27* from the rice cultivar IR24 (gi 66735943 gb AY986492.1). (ii) Sequence of a part of the promoter of rice *Xa10* from the rice cultivar IRBB10 (gi|448280729|gb|JX025645.1|). (iii) A part of the promoters of *Xa23* from the rice cultivar CBB23 (gi|721363841|gb|KP123634.1|) and *xa23* from the rice cultivar JG30 (gi|721363854|gb|KP123635.1|). (iv) A part of the promoters of *Bs3* from *Capsicum annuum* L. cultivar ECW-30R (gi|158851516|gb|EU078684.1) and *Bs3-E* from *C. annuum* L. cultivar ECW (gi|158851512|gb|EU078683.1|). (v) A part of the promoters of *Bs4C-R* from *pubescens* cultivar PI 235047 (gi|414148024|gb|JX944826.1|) and *Bs4C-S* from *Capsicum pubescens* cultivar PI 585270 (gi|414148026|gb|JX944827.1|). The ATG start codon in each case is displayed in red letters. Nucleotides that are identical between the alleles are displayed as black letters. Predicted TATA boxes are underlined. Effector binding elements are highlighted in yellow with blue letters indicating differences between alleles. The TAL effectors are represented by the repeat regions using a single letter represents each RVD (I-NI; G-NG or HG; S-NS; D-HD or ND, *-N*, N-NN). * Represents no amino acid residue at what would otherwise be position 13.

*E* genes are unique in the panoply of *R* genes in that specificity is not in the *R* gene coding sequence but in the expression of the *R* gene in the presence of the effector (Gu et al., 2005; Römer et al., 2007). The TAL effector, itself, contains two notable regions—the central repetitive region and a C-terminal region with NLS motifs and a potent transcription activation domain (AD). The NLS and AD were shown to be required for *E* gene function in the case of *Bs3*, *Xa10*, *Xa7*, and *Xa27* (Van den Ackerveken et al., 1996; Zhu et al., 1998, 1999; Yang et al., 2000; Szurek et al., 2001). TAL effector specificity is determined by the central repetitive region (Herbers et al., 1992; Yang and Gabriel, 1995; Yang and White, 2004; Yang et al., 2005), and is the structural basis for the TAL effector code, where each repeat specifies the probability of accommodating individual nucleotides (Boch et al., 2009; Moscou and Bogdanove, 2009; Deng et al., 2012; Gao et al., 2012; Mak et al., 2012). The repetitive domain consists of 33–35 amino acid repeats that are polymorphic at amino acid residues 12 and 13, which are referred to as the repeat-variable di-residues (RVDs), each of which can be represented by amino acid residue 13 and corresponds to one DNA base in the effector binding element. Proximal to the N-terminal portion of the repetitive domain are non-canonical repeats that mediate pairing with an initial 5′ thymine (Boch et al., 2009; Moscou and Bogdanove, 2009). *E* gene expression occurs upon cognate effector binding to a compatible effector binding element in the respective promoter (Figure 1A). The known *E* genes, with the exception of *Xa10*, have dominant and recessive alleles that differ in DNA sequence polymorphisms in the promoter region (Figure 1B). AvrBs3, for example, fails to induce *Bs3-E*, an allele of *Bs3* with a 13-bp insertion in the effector binding element in the promoter (Figure 1B, iv; Römer et al., 2007, 2009b; Kay et al., 2009). *E* genes, *S* genes, and TAL effector genes, therefore, reflect selective pressures in the evolution of the host and pathogen interaction. In this regard, it is important to note that naturally occurring TAL effectors are not necessarily optimized for the cognate promoters simply in terms of the binding requirements. Natural TAL effector configurations may reflect adaptive responses to other factors, including the level of target gene expression and frequency of binding sites within a genome.

## E Proteins are not Homologs of Classical R Proteins

**FIGURE 2:**
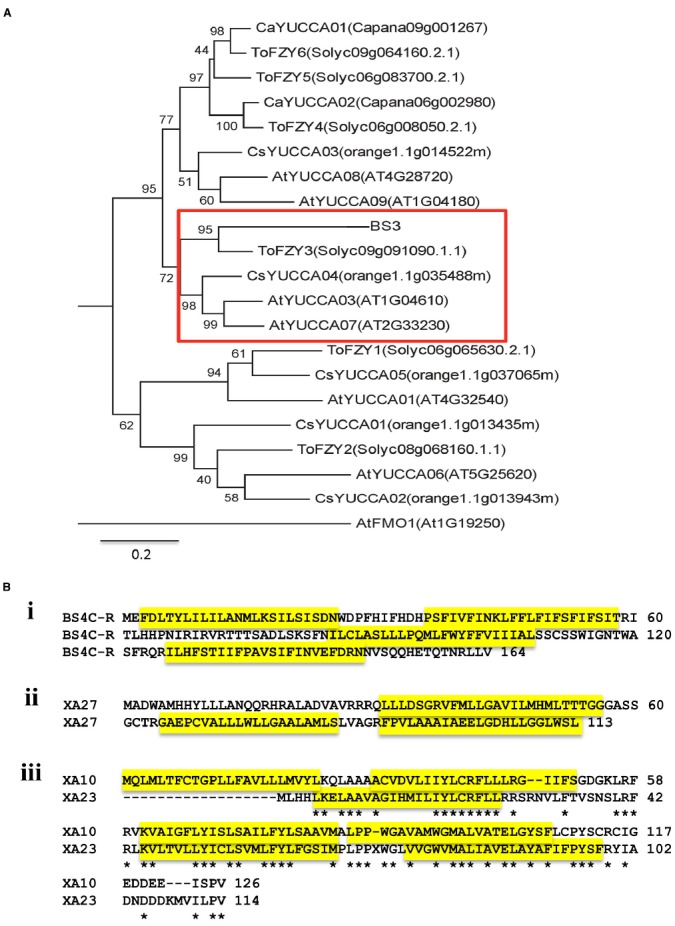
**The known E proteins. (A)** A phylogenetic tree of BS3 related proteins. Proteins from closely related YUCCA proteins of *C. annuum* L. (Ca), *A. thaliana* (At), Tomato (To), *Citrus sinensis* (Cs) and the BS3 protein (Capana02g001306) were aligned. Names of proteins are given with the Phytozome ID or Pepper Genome Database ID (in parentheses). A monophyletic group that contains the predicted BS3 protein and tomato YUCCA-like proteins is boxed in red. Sequences were aligned with the online ClustalW server (http://www.ch.embnet.org/software/ClustalW.html) using the default values. MEGA6.0 was used for generating a tree on the basis of ClustalW output. Phylogenetic calculations are based on the maximum likelihood method, and Bootstrap analysis was used to evaluate the reliability of the nodes of the phylogenetic trees. Bootstrap values are based on 1000 replications. The branch lengths of the tree are proportional to divergence. The 0.1 scale represents 10% change. **(B)** Structural predictions for group 2 E proteins. (i) Bs4C-R; (ii) XA27; (iii) XA10 and XA23. Alignment of XA10 and XA23 was conducted using the online program ClustalW2 using the default parameters (http://www.ebi.ac.uk/Tools/msa/clustalw2/). Transmembrane helices predicted by the SOSUI program (http://bp.nuap.nagoya-u.ac.jp/sosui/sosui_submit.html) are highlighted in yellow. * Represents no amino acid residue at what would otherwise be position 13.

E proteins are not related on the basis of sequence to any other type of R protein. In fact, the proteins, with the exception of the recently reported XA10 and XA23, share no sequence identity with each other. Conceptually, the *E* genes and their products can be divided into two groups. Group 1 consists of proteins that likely have a function in plant development or physiology and whose function has been hijacked by host adaptation to disease. Group 1 consists solely of BS3, which is a member of a conserved family of proteins known as flavin mono-oxygenases (FMO) and, more specifically, a subclass of FMOs known variously as YUCCA or FLOOZY (Figure 2A; Römer et al., 2007; Exposito-Rodriguez et al., 2011; Zhao, 2014). Group 2 members, of which there are four, are relatively short proteins that have multiple hydrophobic potential membrane spanning domains (Figure 2B). The proteins share no sequence relatedness with proteins of known function and the relatively few related coding sequences occur within close relatives. One related sequence outside the *Solanaceae*, from grapevine, was reported for *Bs3C-R*. Several of the E proteins may have structural similarities. XA27 and XA10 are predicted or have been shown to localize to host cellular membranes and XA10, more specifically, has been shown to localize to the endoplasmic reticulum (ER; Wu et al., 2008; Tian et al., 2014). Prediction software also indicates that BS4C-R may be localized to the ER (Nakai and Horton, 1999; Strauss et al., 2012). It is tempting to speculate that BS3 requires catalytic activity for the *R* gene response and the group 2 proteins function as R proteins due to their interaction with host organelles. However, whether the predicted catalytic functions of BS3 are required for the *R* gene response has not been reported, and future analysis of the mechanism-of-action for the respective proteins may indicate some common feature.

## *E* Genes in Bacterial Spot Disease on Pepper

*E* genes for groups 1 and 2 have been cloned from pepper. The group 1 *Bs3* is recognized by both TAL effectors AvrBs3 and AvrHah from the pathogens *Xanthomonas campestris* pv. *vesicatoria* and *Xanthomonas gardneri*, respectively, both causal organisms of bacterial spot disease of pepper and tomato (Bonas et al., 1998; Schornack et al., 2008). The gene product BS3 is a 342 amino acid protein with a high degree of relatedness with FMOs (Römer et al., 2007; Schornack et al., 2008). FMO proteins are a family of enzymes functioning in all phyla (van Berkel et al., 2006), and play roles in pathogen defense, auxin biosynthesis and metabolism of glucosinolates (Bartsch et al., 2006; Koch et al., 2006; Mishina and Zeier, 2006; Schlaich, 2007). As noted earlier, BS3 falls in a phylogenetic clade consisting of YUCCA and ToFZY members (Figure 2A; Römer et al., 2007). The most closely related proteins to BS3 have been demonstrated to be involved in auxin biosynthesis and a variety of developmental and physiological responses (Exposito-Rodriguez et al., 2011; Stepanova et al., 2011; Lee et al., 2012; Hentrich et al., 2013; Zhao, 2014). YUCCA/FLOOZY members catalyze a key intermediate in the plant pathway from indole-3-pyruvate (IPA) into indole-3-acetic acid (IAA) through oxidative decarboxylation reaction (Kim et al., 2011; Stepanova et al., 2011; Dai et al., 2013; Hentrich et al., 2013; Zhao, 2014). A homolog from tomato, *ToFZY*, also functions in auxin biosynthesis (Exposito-Rodriguez et al., 2011). A more distant relative of unknown enzymatic activity, AtFMO1, plays a role in systemic acquired resistance (Mishina and Zeier, 2006).

*Bs4C-R* encodes a member of our group 2 E proteins and is expressed in the presence of the TAL effector AvrBs4 (Strauss et al., 2012). *Bs4C-R* is the only *E* gene isolated on the basis of differential expression between resistant and susceptible cultivars and not the typical gene mapping strategy. A two-nucleotide polymorphism in the region of the effector binding element of a susceptible allele *Bs4C-S* leads to the failure of induction of an AvrBs4-dependent HR (Figure 1B, v; Strauss et al., 2012). Both the dominant and recessive alleles encode functionally competent proteins as constitutive expression of either *Bs4C-R* or *Bs4C-S* triggered HR *Nicotiana benthamiana* in leaves (Strauss et al., 2012).

## *E* Genes in Bacterial Blight Disease of Rice

The *E* genes of rice are all included in our group 2 and provide resistance to bacterial blight disease. Bacterial blight of rice is caused by *Xanthomonas oryzae* pv. *oryzae*, and TAL effectors are major avirulence factors for *X. oryzae* pv. *oryzae* when the cognate *E* genes are present in host plants (Mew, 1987; White and Yang, 2009). Three pairs of TAL effectors and cognate *E* genes have been cloned from rice—AvrXa27/*Xa27*, AvrXa10/*Xa10*, and AvrXa23/*Xa23*. No cognate *S* genes or virulence effects for the TAL effectors of AvrXa10, AvrXa23, or AvrXa27 in compatible host cultivars have been reported, despite the presence of AvrXa27 and AvrXa23 in many extant strains of *X. oryzae* pv. *oryzae* (Gu et al., 2004; Wang et al., 2014).

The *Xa27* product is a protein of 113 amino acids without any clear homologs based on sequence similarity in plants other than rice and several related species of the *Oryza* genus (Gu et al., 2005; Bimolata et al., 2013). The resistance conferred by *Xa27* is affected by developmental stage, increasing with the age of the plants and reaching maximum resistance at 5 weeks. Moreover, *Xa27* showed a dosage effect in the cultivar CO39 genetic background (Gu et al., 2004). At least two transmembrane *α*-helix domains were predicted, depending on the prediction software (Gu et al., 2005). Here, we show three based on the SOSUI program (Figure 2B, Hirokawa et al., 1998). Further experimentation has shown that the protein XA27 localizes to cytoplasmic membrane, and some protein appears in the apoplast after plasmolysis (Wu et al., 2008). Localization is dependent on the N-terminal signal anchor-like sequence, which is also essential for resistance to *X. oryzae* pv. *oryzae* (Wu et al., 2008). The protein itself appears to be toxic as gene transfer to compatible rice lines occurs with a reduced efficiency. Nevertheless recombinant lines were recovered, demonstrating that the AvrXa27-dependency of the resistance is indeed linked to the *Xa27* locus (Gu et al., 2005). At the same time, lines were obtained that had elevated expression of *Xa27* and displayed defense reactions, including thickened vascular elements, even in the absence of bacterial inoculation (Gu et al., 2005). The effector binding element is located immediately downstream of the predicted TATA box, and the recessive allele *xa27* in the susceptible rice cultivar IR24 encodes the same protein but has a three-nucleotide deletion and one nucleotide difference in comparison to *Xa27* (Figure 1B, i; Römer et al., 2009a). DNA sequence alignment of *Xa27* alleles from 27 lines representing four *Oryza* species revealed that a *Xa27*-related coding sequence was indeed present in all of the lines. However, only the IRBB27 allele appears to possess the necessary effector binding element for AvrXa27 (Bimolata et al., 2013). A synthetic TAL effector directed at the recessive allele in IR24 induced a resistance reaction, indicating the product of the recessive allele could function similarly to *Xa27*, if expressed (Li et al., 2013).

*Xa10* encodes a 126-amino acid protein, containing four potential transmembrane helices (Tian et al., 2014). A consensus effector binding element is present in the promoter region of *Xa10* (Figure 1B, iii). *Xa10* differs from *Xa27* and *Bs4C-R* in sequence and by the lack of a nearly identical coding sequence in susceptible plant lines. At the same time, related sequences are found in other lines, including *Xa23* (Wang et al., 2015). Ectopic and weak expression of *Xa10* in rice causes a lesion mimic-like phenotype, while transient expression of *Xa10* in *N. benthamiana* and rice induced HR in plants (Tian et al., 2014). Under the appropriate promoter, *Xa10* also induced programmed cell death (PCD) in mammalian HeLa cells (Tian et al., 2014). In both rice and *N. benthamiana* cells, hydrogen peroxide, swelling and degradation were detected in chloroplasts. Degradation of mitochondria was also observed, supporting the model that XA10 functions as a general inducer of PCD in plant and animal cells (Tian et al., 2014). Further functional characterization revealed that XA10 forms hexamers, localizes on the ER membrane of plant and HeLa cells, and mediates Ca^2+^ depletion, which is consistent with some processes of PCD (Pinton et al., 2008; Williams et al., 2014).

The *E* gene *Xa23* encodes a 113-amino acid protein that shares approximately 50% amino acid sequence identity and 64% nucleotide sequence similarity with XA10 and *Xa10*, respectively (Wang et al., 2015). An identical recessive allele is present on the basis of the coding region, and characterization of the effector binding element of AvrXa23 revealed a 7-bp polymorphism accounts for the failure of *xa23* induction in the recessive rice varieties (Figure 1B, ii). The susceptible cultivar JG30, with *xa23*, became resistant to PXO99^A^ harboring a designed TAL effector specifically targeting the *xa23* promoter region including the 7-bp polymorphism (Wang et al., 2015). Moreover, *Agrobacterium*-mediated transient transformation of *Xa23* indicated that, like *Xa10*, *Xa23* induced an HR in *N. benthamiana*, and also induced an HR in tomato (Wang et al., 2015). Both XA10 and XA23 have a motif of unknown function that is comprised of five acidic amino acid residues (EDDEE and DNDDD, respectively) at the C-termini (Tian et al., 2014). Alteration of the so-called ED motif in XA10 abolished HR activity (Tian et al., 2014).

## Prospects for *E* Genes in Disease Control

The question arises whether, as dominant major genes for resistance, the genes are effective in control of the respective diseases. In rice, only *Xa10* has been deployed in field conditions and is effective against a few extant races of the pathogens (Vera-Cruz et al., 2000; Mishra et al., 2013). *Xa27* has been introduced into breeding programs (Luo et al., 2012; Luo and Yin, 2013). *Xa23* and *Xa7* are also in the process of introduction into various breeding programs (Perez et al., 2008; Huang et al., 2012). But how durable is *E* gene mediated resistance? Bacteria can rapidly evolve to avoid *R* gene recognition through avirulence gene loss under high selection pressure for virulence (Koskiniemi et al., 2012). More specifically, TAL effectors appear to reflect exquisitely the selective forces of evolution in the form of the repetitive domain. Deletion of repeats in AvrBs3, for example, resulted in the loss of the induction of *Bs3* (Römer et al., 2007). Deployment of an *E* gene that targets a critical TAL effector for virulence has been proposed as an approach to make adaptation less likely as the pathogen would have to maintain virulence in addition to losing *Xa7* recognition. Indeed, *Xa7*, which is triggered by the major TAL effector AvrXa7, was found to be durable in field tests in the Philippines (Vera-Cruz et al., 2000). Field isolated strains that arose showed loss of the ability to induce resistance and were weakly virulent presumably due to associated mutations in *avrXa7* (Vera-Cruz et al., 2000). However, *in vitro* rearrangements and *in vivo* selection for loss of AvrXa7-mediated resistance produced gene variants that maintained strain virulence and avoided *Xa7*-mediated resistance (Yang et al., 2005). Furthermore, a number of extant TAL effectors target the same *S* gene as AvrXa7, namely *OsSWEET14*, without activating *Xa7*-dependent resistance (Antony et al., 2010; Yu et al., 2011; Streubel et al., 2013). Strains can also acquire other major TAL effectors that target alternative paralogs of that *S* gene (Yang et al., 2006; Streubel et al., 2013; Zhou et al., 2015).

In India, field strain surveys have found a diversity of strains, many without AvrXa7 activity, indicating that any benefits in the deployment of *Xa7* would be short-lived (Mishra et al., 2013). Thus, broad application of a single *E* gene like *Xa7* in some environments appears to be limited. At the same time, local conditions, such as in the Philippine tests, may limit the invasion of a particular TAL effector gene into extant pathogen populations and deployment may be both broad and durable (Vera-Cruz et al., 2000). *Xa27* and *Xa23* are interesting from the perspective that the cognate avirulence genes are present in many strains and, therefore, broadly effective (Gu et al., 2004; Wang et al., 2014). In contrast to AvrXa7, no cognate *S* genes have been reported for AvrXa27 or AvrXa23, so we can speculate that these effectors may provide some fitness to the pathogen which has not been detected in laboratory or greenhouse assays so far. AvrBs3 has a phenotypic effect for strains of *Xanthomonas euvesicatoria* harboring the gene; a fitness benefit for the effector has been observed (Marois et al., 2002; Wichmann and Bergelson, 2004), and *Bs3* is effective for many pepper strains of *X. euvesicatoria*. In addition, *Bs3* is also effective against the emerging pepper pathogen *X. gardneri*, which harbors the TAL effector AvrHah1 (Schornack et al., 2008; Schwartz et al., 2015).

## *E* Genes and New Strategies for Resistance

Despite possible shortcomings of endogenous *E* genes, *E* genes hold great potential for breeding broadly and durably resistant crop varieties. Specifically for TAL effector associated diseases, *E* genes can be constructed with so-called super-promoters, consisting of multiple effector binding sites, each recognizing specific corresponding TAL effectors that are expressed in the pathogen populations (Römer et al., 2009a; Hummel et al., 2012; Zeng et al., 2015). *Xa27* was fused to a super promoter including binding sites for three TAL effectors from *X. oryzae* pv. *oryzae* and three from the bacterial leaf streak pathogen *X. oryzae* pv. *oryzicola*. The plants were resistant to several *X. oryzae* pv. *oryzae* and *X. oryzae* pv. *oryzicola* strains that were originally compatible on wild type homozygous *Xa27* plants (Hummel et al., 2012). Similarly, transgenic rice lines containing *Xa10^E5^* with binding elements to five TAL effectors proved to be resistant to 27 of the 28 selected *X. oryzae* pv. *oryzae* strains gathered from 11 countries (Zeng et al., 2015). Judicial choices of the effector binding sites for TAL effectors in extant populations may provide resilient barriers to TAL effector associated diseases. However, due to the risk that an added effector binding element might coincidently contains a *cis* regulatory element which could induce the *E* gene expression in response to particular stimuli and cause cell death without challenge of TAL effectors, such amended promoters should be tested thoroughly before deployment (Hummel et al., 2012). Another approach is to engineer an *E* gene to be under the control of a different type of pathogen inducible promoter. For example, expression of *Xa27* under the control of an disease inducible or defense gene promoter, in this case, the rice PR1 promoter, which is induced by both compatible and incompatible bacteria, conferred broad resistance to *X. oryzae* pv. *oryzae* strains (Gu et al., 2005). This strategy need not be limited to *Xanthomonas* related diseases.

## Conclusion

Thirteen *R* genes have been cloned for resistance to *Xanthomonas* diseases—all coming from rice, pepper, or tomato. Four, in addition to *Bs4*, are representatives of the two major classes of *R* genes, the receptor linked kinases (*RLK*) and nucleotide binding site leucine rich repeat (*NBS-LRR*) genes which are represented by *Xa21* (*RLK*, rice), *Xa26* (*RLK*, rice), *Xa1* (*NBS-LRR*, rice), and *Bs2* (*NBS-LRR*, pepper; Yoshimura et al., 1998; Tai et al., 1999; Zhang and Wang, 2013). Three cloned genes are recessive genes from rice and, although not discussed here, can be considered cases of loss of susceptibility (White and Yang, 2009). The five *E* genes and the protein products bear little or no resemblance to the other *R* genes, or, for that matter, other common defense response components. *E* genes, at least phenotypically, trigger host responses, in particular the HR, similarly to some other *R* gene mediated resistances. Whether the E proteins intersect other R protein mediated resistance pathways in plants remains unknown. Evidence for XA10 indicates that the protein activates PCD, possibly through ER-stress in light of the association with the ER (Williams et al., 2014). Further research into *E* gene functions should enhance their utility for new resistance strategies as well as improve our understanding on plant defense and PCD pathways.

### Conflict of Interest Statement

The authors declare that the research was conducted in the absence of any commercial or financial relationships that could be construed as a potential conflict of interest.
